# WormNet v3: a network-assisted hypothesis-generating server for *Caenorhabditis elegans*

**DOI:** 10.1093/nar/gku367

**Published:** 2014-05-09

**Authors:** Ara Cho, Junha Shin, Sohyun Hwang, Chanyoung Kim, Hongseok Shim, Hyojin Kim, Hanhae Kim, Insuk Lee

**Affiliations:** 1 Department of Biotechnology, College of Life Science and Biotechnology, Yonsei University, Seoul, Korea; 2 Center for Systems and Synthetic Biology, University of Texas at Austin, Austin, TX, USA

## Abstract

High-throughput experimental technologies gradually shift the paradigm of biological research from hypothesis-validation toward hypothesis-generation science. Translating diverse types of large-scale experimental data into testable hypotheses, however, remains a daunting task. We previously demonstrated that heterogeneous genomics data can be integrated into a single genome-scale gene network with high prediction power for ribonucleic acid interference (RNAi) phenotypes in *Caenorhabditis elegans*, a popular metazoan model in the study of developmental biology, neurobiology and genetics. Here, we present WormNet version 3 (v3), which is a new network-assisted hypothesis-generating server for *C. elegans*. WormNet v3 includes major updates to the base gene network, which substantially improved predictions of RNAi phenotypes. The server generates various gene network-based hypotheses using three complementary network methods: (i) a phenotype-centric approach to ‘find new members for a pathway’; (ii) a gene-centric approach to ‘infer functions from network neighbors’ and (iii) a context-centric approach to ‘find context-associated hub genes’, which is a new method to identify key genes that mediate physiology within a specific context. For example, we demonstrated that the context-centric approach can be used to identify potential molecular targets of toxic chemicals. WormNet v3 is freely accessible at http://www.inetbio.org/wormnet.

## INTRODUCTION

*Caenorhabditis elegans* has many advantages, such as genetic manipulability, as a model organism for the study of development, neuroscience, and other complex metazoan phenotypes. The study of *C. elegans* has provided numerous insights for human disease research because many human disease pathways have been conserved in *C. elegans* (1). Mapping gene-to-phenotype associations is widely considered to be the first step toward understanding the genetic organization of such phenotypes (2). Testing loss-of-function phenotypes has been a major approach to mapping gene-to-phenotype associations. Gene loss-of-function can be tested by either the disruption of coding deoxyribonucleic acid (DNA) (knockout) or the inhibition of messenger ribonucleic acid (mRNA) translation (knockdown). *Caenorhabditis elegans* has been a favored model in animal genetics research due to an efficient knockdown protocol based on RNA interference (RNAi) (3). In addition, recently developed CRISPR-Cas9 systems enable high-throughput gene knockouts in *C. elegans* (4). Testing all ∼20 000 genes of the *C. elegans* genome, however, is expensive and may require years of screening experiments with potentially many false negatives. Bioinformatics tools to prioritize candidate genes or phenotypes are therefore highly desired. 

Gene networks are useful for identifying novel genes that are associated with specific phenotypes, including for human diseases, because genes for the same loss-of-function phenotypes (e.g. diseases) tend to be proximal in a co-functional network (2,5,6). Network-assisted hypothesis generation has proven effective in the identification of genes associated with phenotypes in *C. elegans*. The gene network model, WormNet (7,8), and network-assisted prediction methods have been previously implemented as a web server. Since the publication of the last version of the WormNet web server, WormNet version 2 (v2) (8), major updates to publicly available genomics data as well as algorithms for mapping co-functional gene links have occurred. For example, while WormNet v2 contains co-expression links derived mainly from low quality spotted microarray platforms, the publicly available Gene Expression Omnibus (GEO) database (9) currently contains more than 1600 *C. elegans* expression profiles derived from Affymetrix DNA chips, a platform that provides more statistically controllable data. An update of WormNet to incorporate these new data and algorithms would therefore further improve the prediction power of our network-assisted prediction server.

Here, we present the web server, WormNet v3, which updates the base gene network as well as the prediction methods from previous versions. The updates in WormNet v3 substantially improve the prediction power for RNAi phenotypes. A new prediction method, ‘find context-associated hub genes’, that can identify key player genes that mediate physiology within a particular biological context, including chemical intoxication, is also introduced in WormNet v3.

## UPDATES TO THE BASE GENE NETWORK

The base gene network for our network-assisted prediction server is constructed by training heterogeneous genomics data using machine learning techniques. The prediction power of this server is determined mainly by the quality of the base gene network. There are three major components that influence the quality of gene networks constructed by machine learning approaches: the training data, the raw input data and the linkage mapping algorithms. We have revised all three components in WormNet v3. These changes are summarized in Supplementary Table S1; a few of these changes are highlighted below.

To develop the training data for WormNet v3, we excluded gene pairs that share gene ontology biological process (GO-BP) terms based on the IMP (inferred from mutant phenotype) evidence code. Many GO-BP annotations for *C. elegans* genes have been inferred from mutant phenotypes. We noticed that most GO-BP terms for *C. elegans* phenotypes are related to the organism-level morphology. We presumed that identical organism-level morphology may result from perturbations of genes in unrelated molecular pathways. For example, defects in embryo development, larval development, growth, reproduction, locomotion and body morphogenesis may result from dysfunctions in functionally unrelated molecular pathways. Pairing genes by mutant phenotypes would therefore generate many between-pathway links. Because the ultimate aim is to reconstruct molecular pathways via co-functional gene networks, these gene pairs that share GO-BP terms based on IMP were excluded from the training data for the new gene network. Using this modification on the training data, we generated 78 739 positive and 2 909 054 negative gold standard gene pairs. The likelihood scores for co-functional links between genes were calculated using a Bayesian statistics approach in which each link was assigned a log likelihood score (LLS) as for the previous network (7).

The most notable update to the raw input data is the use of new gene expression data for the co-expression networks. Over the past several years, a large amount of expression data has been generated by commercial DNA chips, which provide more robust signals and more sophisticated statistical analysis packages. We analyzed 34 expression sets that contained no less than 10 gene expression samples (862 samples in total) from Affymetrix DNA chips (GPL200 platform of GEO) and constructed co-expression networks as for the previous network (7) from 12 sets containing 456 samples in total (GSE numbers are listed in Supplementary Table S1). Similar co-expression networks were constructed for *Saccharomyces cerevisiae, Drosophila melanogaster*, *Danio rerio* and *Homo sapiens*, and these co-expression links were transferred to *C. elegans* by orthology (10). The number of protein–protein interactions in the raw input data also was increased significantly due to the improved databases and newly reported large-scale interaction data (summarized in Supplementary Table S1). We also improved methods for linkage mapping based on gene neighborhood (11) and phylogenetic profiles (12) as described in the Supplementary Online Methods.

**Figure 1.**
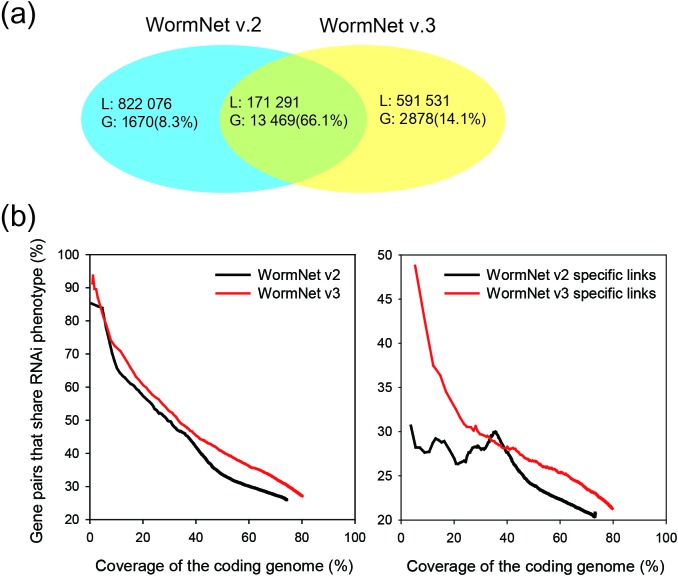
(**a**) A Venn diagram of genes (the percentage coverage of the coding genome is indicated in the parenthesis) and links in WormNet v2 and WormNet v3. (**b**) A comparison of the prediction power between WormNet v2 and WormNet v3 using a total of 478 RNAi phenotypes, which contain between 5 and 500 genes, collected from WormBase239 (13). Network precision was measured by calculating the percentage of gene pairs that share RNAi phenotypes for different coverage of the coding genome. WormNet v3 shows superior performance over the entire range of the genome coverage. The assessment of new links in WormNet v3 (WormNet v3 specific links) and the excluded old links (WormNet v2 specific links) confirmed that the improved precision of WormNet v3 is attributable to the new network links that have been included in this update.

A total of 19 different data types derived from five different species were integrated by a weighted sum method as for the previous network (7), which resulted in a gene network of 762 822 links that cover 16 347 *C. elegans* genes [i.e. 80.2% of the 20 389 coding genome in WormBase220 (13)]. The list of edges in the integrated network as well as details about all 19 individual networks, which have been derived from different data types, are available for download from the ‘network download’ page. Compared with the previous gene network, the genome coverage of the new network increased from 74.5 to 80.2% (1208 additional genes) while the number of network links was reduced. We also found that 171 291 links and 13 469 genes were common between WormNet v2 and v3, 822 076 links and 1670 genes from WormNet v2 were excluded from WormNet v3 and 591 531 new links and 2878 new genes were added to WormNet v3 (Figure 1a). To test whether these changes improved the overall prediction power of our server, we measured network precision by computing the proportion of the network gene pairs that share the same RNAi phenotypes for different coding genome coverage. We used a total of 478 RNAi phenotype sets, which contained between 5 and 500 genes, collected from WormBase239 (13). From this assessment, we found that the network precision is significantly improved in WormNet v3 and that this improvement is largely attributable to the new links included in WormNet v3 (Figure 1b). This large change in network links but not in prediction power may be explained by the fact that pathway genes can remain well-connected by different sets of links.

## NETWORK-ASSISTED PREDICTION METHODS

**Figure 2.**
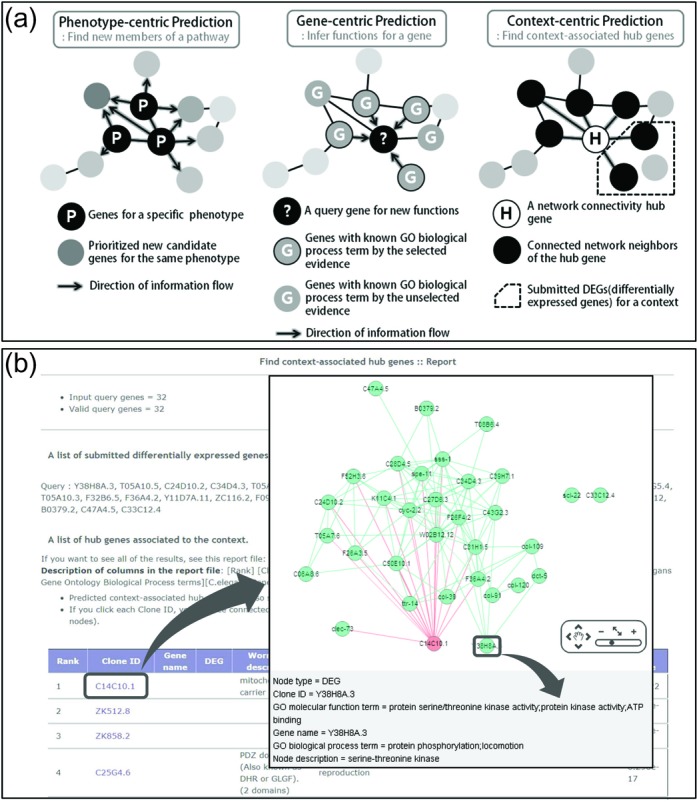
(**a**) A schematic illustration of the three network-assisted prediction methods. (**b**) Screen shots of the prediction results that are returned by the ‘find context-associated hub genes’ method. The analysis returned a table of hub genes that are predicted to be associated with the biological context characterized by the submitted differentially expressed genes (DEGs). If a user clicks a candidate hub (e.g. C14C10.1 shown in the table), a new web page displays the network of the hub gene and its neighbors. The network shows all links among the submitted DEGs as well as links from the hub to its neighbors that overlap with the given DEGs. By clicking a node or an edge of the network, users can also view in the lower panel detailed information about the gene or the co-functional link.

The WormNet v3 server generates new hypotheses using three complementary network methods, which are illustrated in Figure 2a. For each prediction method, WormNet v3 server provides a toy example for a test run. The first method, ‘find new members for a pathway’, is a phenotype-centric method. This approach predicts new candidate genes for a phenotype using known genes for that phenotype, namely seed genes, which are submitted by the user. The server returns the top 200 ranked candidate genes for the phenotype of interest using the sum of the network edge weights (i.e. the LLS) on all the submitted seed genes. The WormNet v3 server also reports the network prediction power for the submitted seed genes using a receiver operating characteristic (ROC) curve, the results of which are summarized as a single score, the area under the ROC curve (AUC). Perfect prediction power results in an AUC equal to 1 and predictions that represent random chance result in an AUC equal to 0.5. Generally, an AUC that is >0.7 indicates good prediction power. If a high AUC is observed for the submitted seed genes, then the predicted candidate genes are more likely to show the mutant phenotype upon perturbation.

The second prediction method, ‘infer functions from network neighbors’, is a gene-centric approach that predicts GO-BP functional terms for a query gene that is submitted by the user. The server collects all annotated GO-BP terms for the query gene from connected network neighbors and ranks the GO-BP terms using the sum of the network edge weights (i.e. the LLS) on genes annotated by each GO-BP term. The server returns the top 10 GO-BP terms as candidate functions for the query gene.

These two methods existed in previous versions of WormNet. A new network prediction method has been incorporated in WormNet v3. This method is based on a context-centric approach, ‘find context-associated hub genes’ that can predict important genes for a given biological context. For this analysis, the server uses pre-defined subnetworks, which are composed of a hub gene and its connected neighbors. These hub genes are hubs for each of the subnetworks, not for the whole gene network. In the new base gene network, we considered only subnetworks for hub genes that have no >15 neighbors connected by LLS > 1, which resulted in 7025 hub genes for the subsequent analyzes. Users initiate a prediction by submitting a set of differentially expressed genes (DEGs) that characterize the biological context. For example, the DEGs of *C. elegans* that have been exposed to toxic chemicals can characterize the context of intoxication for the organism. The server measures the association between the hub genes and the context by statistical enrichment of the hub's neighbors among the submitted DEGs using Fisher's exact test, and returns all hub genes that are significantly associated with the context (Figure 2b). In addition, the expression level of some of the important genes for a particular biological context may change. Therefore, the context-centric prediction method often ranks DEGs highly, which highlights the ability of this method to predict important genes. A more detailed description of the concepts underlying the context-centric prediction method is provided in the Supplementary Online Methods (Supplementary Figure S1).

## CASE STUDIES

**Figure 3.**
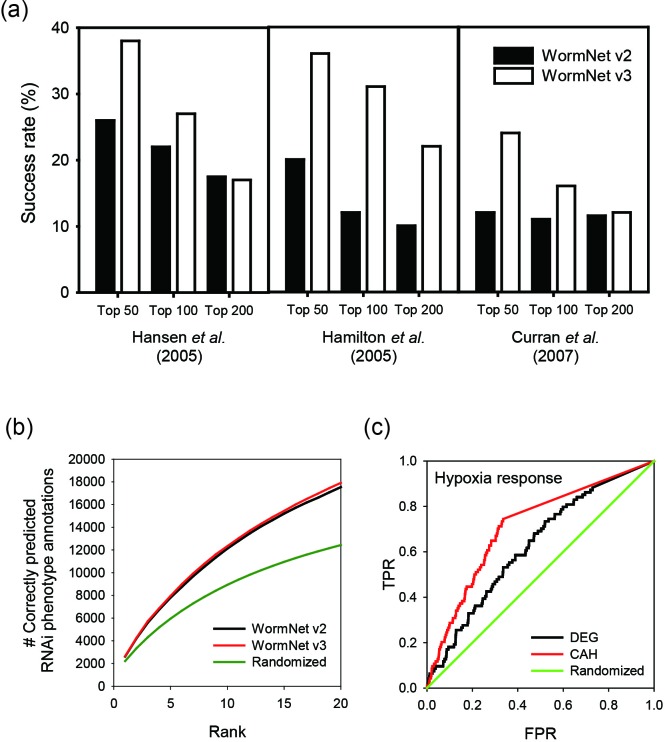
(**a**) A bar graph that shows the success rates of the predictions for the ‘extended life span’ genes. To assess the effectiveness of the phenotype-centric prediction, we performed a simulation in which 372 genes for ‘extended life span’ collected from WormBase239 (13) were predicted by network connectivity to the seed genes derived from each of three independent genome-wide RNAi screens: 29 genes from Hansen *et al*. (14), 85 genes from Hamilton *et al*. (15) and 61 genes from Curran *et al*. (16). For each seed gene set, the WormNet server prioritized new candidate genes for extended life span. The efficiency of each prediction was measured by the success rate, which was computed as the percentage of recapitulated non-seed genes of the 372 known genes for extended life span among the top 50, 100 and 200 candidates. The success rates ranged from 24 to 38% among the top 50 candidates for the three query sets. This range was slightly lower among the top 100 or 200 candidates. Notably, the success rate was significantly reduced when the base gene network in WormNet v2 was used, which demonstrates the significant improvement in network quality in WormNet v3. (**b**) A performance is measured by the number of correctly predicted RNAi phenotype annotations (y-axis) for the given rank threshold (x-axis). WormNet v3 performs slightly but consistently better than WormNet v2. Both versions of WormNet outperform randomized predictions (the curve represents the average performance of 100 random predictions). (**c**) A ROC curve that shows high performance of the context-centric prediction method for hypoxia response for associated genes annotated by the RNAi phenotype. Predictions based on context-associated hub (CAH) genes outperform those based on DEGs. TPR, true positive rate; FPR, false positive rate; randomized, random prediction.

To demonstrate the feasibility of useful hypothesis generation by the three network-assisted methods in WormNet v3, we performed a case study for each prediction method with query genes as toy examples in the server. First, we simulated the prediction of 372 genes for ‘extended life span’ collected from WormBase239 (13) using the phenotype-centric method. For this simulation, we submitted genes for life span extension that have been identified from genome-scale RNAi screens by Hansen *et al*. (29 genes) (14), Hamilton *et al*. (85 genes) (15) and Curran *et al*. (61 genes) (16) to the server, and then measured the success rate of the predictions as the percentage of recapitulated non-seed genes from the 372 known genes among the top candidates. We found that the success rates ranged from 24 to 38% among the top 50 candidates for the three query sets; this success rate was slightly lower among the top 100 or 200 candidates (Figure 3a). Given that the random discovery rate for these 372 genes from the pool of 20,389 genes is less than 2% (372/20 389 = 1.82%), this network-assisted prediction in WormNet v3 achieved a more than 10-fold enrichment. In addition, the success rate on this same set of 372 genes was significantly reduced when the base network in WormNet v2 was used, which confirms the improved quality of the base network in WormNet v3.

Next, we systematically assessed the gene-centric prediction method. In WormNet v3, this method is designed to predict GO-BP terms. To test the predictive power of this method, however, we used RNAi phenotype annotations, which are independent from the GO-BP annotations that were used for the original training of WormNet. We collected 42 831 annotations for 505 RNAi phenotypes from 6743 genes in WormBase239 (13). We used a leave-one-out analysis method in which the known RNAi phenotype annotations of a gene were masked and then newly predicted by the enriched RNAi phenotypes among its network neighbors for each round of simulated prediction. A total of 17 915 known gene-phenotype associations (42% of all known associations) were correctly predicted within the top 20 predicted phenotypes (Figure 3b). We performed the same analysis for WormNet v2 (8) as well as 100 randomized networks, and found that performance was improved in WormNet v3 compared with WormNet v2 (17 539, 41%) and the randomized networks (12 432, 29%).

The effectiveness of the gene-centric prediction method was also demonstrated using recently updated GO-BP annotations. WormNet v3 uses the GO-BP annotations that were downloaded on November 2011. Since November 2011, many new GO-BP annotations have been added to *C. elegans* genes. We found that 169 genes have been newly annotated by GO-BP terms with reliable GO evidence codes (IDA, inferred from direct assay; IMP, inferred from mutant phenotype; IGI, inferred from genetic interaction; IPI, inferred from physical interaction; IEP, inferred from expression pattern; TAS, traceable author statement; ISS, inferred from sequence or structural similarity) and that 42 of these genes were correctly predicted as top 10 candidate functions (∼25% success rate). These successful predictions can be demonstrated by running toy examples of 11 genes that were newly annotated by the GO-BP term for ‘reproduction’ after November 2011, which were correctly predicted as top10 candidates by WormNet v3.

Finally, we tested a new context-centric prediction method, ‘find context-associated hub genes’, using toxicogenomics data derived from the exposure of *C. elegans* to the organophosphate pesticide, dichlorvos (17). The principle mechanism of acute toxicity by organophosphate pesticide is the inhibition of acetylcholinesterase. Molecular mechanisms for the observed persistent and delayed toxic effects, however, have remained largely unknown. We hypothesized that an important gene for dichlorvos intoxication will be functionally connected with many DEGs upon exposure to the pesticide. By testing DEGs that have been detected from prolonged exposure to a low concentration of dichlorvos, the hub genes that are tightly connected to the context-associated DEGs may emerge as target genes that mediate the delayed toxic effect. We therefore conducted an analysis with 32 up-regulated genes (defined by >1.5-fold increase of expression levels after 26 h) and identified gene C14C10.1, which is a putative mitochondrial carrier protein, as the top candidate gene associated with prolonged intoxication by dichlorvos. Mitochondrial dysfunction has been suggested as a mechanism of intoxication (17) and many human diseases, such as metabolic disorders, neurodegenerative diseases, and muscle dystrophy, are associated with mutations of the mitochondrial carrier proteins (18). C14C10.1 may therefore represent a potential target for dichlorvos. Taken together, these results suggest that WormNet v3 can predict target genes for a chemical when appropriate toxicogenomics data are used as input. We provided the 32 DEGs used in this case study as a toy example to simulate the context-centric prediction. Given the increasing use of *C. elegans* in toxicology (19), this context-centric prediction of WormNet v3 may prove to be useful in the identification of potential targets or key modulators for intoxication in the study of many toxic chemicals.

A more quantitative assessment of the context-centric prediction method was performed using three contexts for which both genome-wide expression data from GEO (9) and RNAi phenotype annotations are available: hypoxia response, heat response and dauer development (see Supplementary Online Methods for detailed descriptions). To generate a set of DEGs for each context, we ranked genes by the expression change compared with control experiments, and collected the top 200 genes for each context. We then calculated probability of context-association of a gene for each context using corresponding 200 DEG sets. Finally, genes were prioritized for each context by either probability of expression change for the context (i.e. DEG) or probability of being context-associated hub (i.e. CAH). Genes relevant to each context as annotated by the RNAi phenotypes were used to measure the true positive rate and false positive rate of the ROC curve analysis (Figure 3c and Supplementary Figure S2). For all three tested contexts, CAH outperformed DEG in retrieving genes known to be associated with the context by RNAi phenotypes.

## SUMMARY

WormNet v3 is a new network-assisted hypothesis-generating server for *C. elegans*. Both the base gene network and the prediction methods have been updated from previous versions. The improved quality of the base gene network was validated by testing the prediction of RNAi phenotypes. In addition to the two pre-existing prediction methods, ‘find new members for a pathway’ and ‘infer functions from network neighbors’, a new context-centric prediction method, ‘find context-associated hub genes’, was added to WormNet v3. This new method may be useful in the study of molecular mechanisms of intoxication given related toxicogenomics data. WormNet v3 therefore provides a comprehensive network-assisted prediction platform with three complementary approaches to facilitate genetic dissections of complex phenotypes in *C. elegans*.

## SUPPLEMENTARY DATA

Supplementary Data are available at NAR Online.

## FUNDING

National Research Foundation of Korea [2010-0017649, 2012M3A9B4028641, 2012M3A9C7050151]; Next-Generation BioGreen 21 Program [SSAC, PJ009029 to I.L.]. Funding for open access charge: National Research Grant.

*Conflict of interest statement*. None declared.

## Supplementary Material

Supplementary Data
