# Supplementary material for: WormNet v3: a network-assisted hypothesis-generating server for Caenorhabditis elegans
Source: Nucleic Acids Res. 2014 May 9;42(Web Server issue):W76–82. doi: 10.1093/nar/gku367 (PMC4086142; doi:10.1093/nar/gku367)
Supplement: Supplementary Data [file supp_42_W1_W76__index.html]

Supplementary Data 

# WormNet v3: a network-assisted hypothesis-generating server for *Caenorhabditis elegans*

## Supplementary Data

**Files in this Data Supplement:**

- SUPPLEMENTARY DATA
- SUPPLEMENTARY DATA
