# Supplementary material for: WormNet v3: a network-assisted hypothesis-generating server for Caenorhabditis elegans
Source: Nucleic Acids Res. 2014 May 9;42(Web Server issue):W76–82. doi: 10.1093/nar/gku367 (PMC4086142; doi:10.1093/nar/gku367)
Supplement: Supplementary Data [file supp_gku367_nar-00499-web-b-2014-File006.docx]

**Supplementary Online Methods**

**WormNet v3: a network-assisted hypothesis-generating server for *Caenorhabditis elegans***

Ara Cho, Junha Shin, Sohyun Hwang, Chanyoung Kim, Hongsuk Shim, Hyojin Kim, Hanhae Kim, and Insuk Lee

**Links inferred from phylogenetic profile similarity and gene neighborhood**

Evolutionary and regulatory constraints on functional couplings between genes result in similar genomic contexts. We used the two most effective genomic context approaches to infer co-functional links from publicly available genome sequences: phylogenetic profile similarity (PG) ([1-3](#_ENREF_1)) and genomic neighborhood (GN) ([4-6](#_ENREF_4)). To construct WormNet v3, we used a total of 2,144 sequenced genomes (122 from Archaea, 1,626 from Bacteria, and 396 from the Eukaryota domain).

The phylogenetic profile similarity between two worm genes indicates the degree of co-inheritance during speciation. To identify functional links between worm genes, we first ran PBLAST to compare all worm protein sequences against all protein sequences from the 2,144 genomes. Phylogenetic profile matrices were constructed from the blast hit scores, and the similarity between profiles was measured by mutual information scores as described in Date *et al.* ([7](#_ENREF_7)). In contrast to the previous method, which uses the similarity of phylogenetic profiles based on all genomes, the new method uses domain-specific phylogenetic profiles. Recently, we found that the similarity of phylogenetic profiles for each of the three domains of life (Archaea, Bacteria, and Eukaryota) performed better than monolithic profiles based on all 2,144 genomes in retrieving gold-standard co-functional links. For worm genes, we found a high retrieval rate of gold standard co-functional links with the Archaea-specific profiles and the Bacteria-specific profiles, but not with the Eukaryota-specific profiles. Therefore, for the phylogenetic profile method, we constructed two networks for each of the two domains and then integrated the two networks into a single final network.

There are two approaches to measure the genomic neighborhood: the chromosomal distance between neighboring genes ([5](#_ENREF_5),[6](#_ENREF_6),[8](#_ENREF_8)) and the probability of the observed neighborhood ([4](#_ENREF_4)). Recently, we found that these two methods infer complementary links for similar sets of genes ([9](#_ENREF_9)). For WormNet v3, we constructed one network using the distance-based method and another network using the probability-based method, and then integrated the two networks into a single final network.

**Context-centric network prediction and quantitative assessment**

In many functional genomics studies, differentially expressed genes (DEGs) are considered to be important genes associated with adaptation to a given cellular or organismal context. Gene expression changes often result, however, from indirect effects due to changes in key genes that are more directly associated with the context. For this analysis, therefore, we assumed that key genes that affect the expression of many other genes also tend to be functionally coupled with these genes. We extracted subnetworks composed of a gene and its network neighbors from WormNet, and considered only genes with no less than 15 neighbors connected by a LLS > 1. The resultant subnetworks are star topology networks in which each network is centralized by a hub gene representing functional relationships between a primary gene and many other genes. If the hub of a subnetwork is primarily associated with a given context, its neighbors may be enriched by DEGs associated with the same context. Therefore, we can infer an association between the hub gene and the context by the association between the hub’s neighbor genes and context-specific DEGs. We measure the significance of the association between two gene sets using Fisher’s exact test. If their association is statistically significant (i.e., p-value < 0.01), we consider the hub gene to be a ‘context-associated hub (CAH)’ gene. We expect that CAHs are more directly associated with the given context than the original DEGs. The concepts underlying this context-centric prediction method are schematically illustrated in **Supplementary Figure 1**.

To quantitatively assess the prediction power of the context-centric prediction method, we used three different contexts for which both genome-wide expression data from Gene Expression Omnibus (GEO) ([10](#_ENREF_10)) and RNAi phenotype annotations are available. We used three GEO series: GSE2836 for hypoxia response, GSE945 for heat response, and GSE30977 for dauer development. We also used the following worm phenotype ontologies for collecting RNAi phenotypes from WormBase239 ([11](#_ENREF_11)): (1) WBPhenotype:0001663 (organism oxidative stress resistant), WBPhenotype:0001621 (organism oxidative stress response hypersensitive), WBPhenotype:000464 (oxygen response variant), and WBPhenotype:0001989 (hypoxia hypersensitive) for the hypoxia response context; (2) WBPhenotype:0001273 (organism heat response variant) and WBphenotype:0001274 (organism heat hypersensitive) for the heat response context; and (3) WBPhenotype:0000012 (dauer constitutive), WBPhenotype:0000637 (dauer formation variant), WBPhenotype:0000308 (dauer development variant), WBPhenotype:0001545 (dauer body morphology variant), WBPhenotype:0000159 (dauer arrest variant), and WBPhenotype:0001539 (dauer induction variant) for the dauer development context.

**References**

1. Pellegrini, M., Marcotte, E.M., Thompson, M.J., Eisenberg, D. and Yeates, T.O. (1999) Assigning protein functions by comparative genome analysis: protein phylogenetic profiles. *Proc Natl Acad Sci U S A*, **96**, 4285-4288.

2. Huynen, M., Snel, B., Lathe, W., 3rd and Bork, P. (2000) Predicting protein function by genomic context: quantitative evaluation and qualitative inferences. *Genome Res*, **10**, 1204-1210.

3. Wolf, Y.I., Rogozin, I.B., Kondrashov, A.S. and Koonin, E.V. (2001) Genome alignment, evolution of prokaryotic genome organization, and prediction of gene function using genomic context. *Genome Res*, **11**, 356-372.

4. Bowers, P.M., Pellegrini, M., Thompson, M.J., Fierro, J., Yeates, T.O. and Eisenberg, D. (2004) Prolinks: a database of protein functional linkages derived from coevolution. *Genome biology*, **5**, R35.

5. Dandekar, T., Snel, B., Huynen, M. and Bork, P. (1998) Conservation of gene order: a fingerprint of proteins that physically interact. *Trends Biochem Sci*, **23**, 324-328.

6. Overbeek, R., Fonstein, M., D'Souza, M., Pusch, G.D. and Maltsev, N. (1999) The use of gene clusters to infer functional coupling. *Proc Natl Acad Sci U S A*, **96**, 2896-2901.

7. Date, S.V. and Marcotte, E.M. (2003) Discovery of uncharacterized cellular systems by genome-wide analysis of functional linkages. *Nature biotechnology*, **21**, 1055-1062.

8. Korbel, J.O., Jensen, L.J., von Mering, C. and Bork, P. (2004) Analysis of genomic context: prediction of functional associations from conserved bidirectionally transcribed gene pairs. *Nature biotechnology*, **22**, 911-917.

9. Shin, J., Lee, T., Kim, H. and Lee, I. (2014) Complementarity between distance- and probability-based methods of gene neighbourhood identification for pathway reconstruction. *Mol Biosyst*, **10**, 24-29.

10. Barrett, T., Wilhite, S.E., Ledoux, P., Evangelista, C., Kim, I.F., Tomashevsky, M., Marshall, K.A., Phillippy, K.H., Sherman, P.M., Holko, M. *et al.* (2013) NCBI GEO: archive for functional genomics data sets--update. *Nucleic Acids Res*, **41**, D991-995.

11. Schindelman, G., Fernandes, J.S., Bastiani, C.A., Yook, K. and Sternberg, P.W. (2011) Worm Phenotype Ontology: integrating phenotype data within and beyond the C. elegans community. *BMC bioinformatics*, **12**, 32.

**Supplementary Figure 1.**

A schematic illustration of the concepts underlying context-centric network prediction.

**Supplementary Figure 2.**

Receiver operating characteristic (ROC) analysis results for predicted genes in a context using differentially expressed genes (DEG) or context-associated hubs (CAH). CAH outperformed DEG in retrieving genes known to be relevant to heat response (left panel) or dauer development (right panel) by RNAi phenotype. TPR, true positive rate; FPR, false positive rate; Randomized, random prediction
