# Supplementary material for: WormNet v3: a network-assisted hypothesis-generating server for Caenorhabditis elegans
Source: Nucleic Acids Res. 2014 May 9;42(Web Server issue):W76–82. doi: 10.1093/nar/gku367 (PMC4086142; doi:10.1093/nar/gku367)
Supplement: Supplementary Data [file supp_gku367_nar-00499-web-b-2014-File005.docx]

**Table S1. Comparisons between WormNet v2 and v3**

|  | **WormNet version 2** | **WormNet version 3** |
| --- | --- | --- |
| Network genes | 20,081 coding genes from WormBase170 | 20,389 coding genes from WormBase220 ([1](#_ENREF_1)) |
| Gene pairs for network training | Gene pairs that share the same Gene Ontology ([2](#_ENREF_2)) biological process annotations as supported by all GO evidence codes on March 2005 | Gene pairs that share the same Gene Ontology ([2](#_ENREF_2)) biological process annotations as supported by GO evidence codes for ISS, IDA, TAS, IPI on September 2011, or the same KEGG (Kyoto Encyclopedia of Genes and Genomes) ([3](#_ENREF_3)) PATHWAY annotations as of March 2012. |
| Network Coverage | 999,367 links among 15,139 genes (74.5% of annotated genes in WormBase170) | 762,822 links among 16,347 genes (80.2% of annotated genes in WormBase220) |
| CE-CX | Integrated co-expression links from 7 sets of experiments using in-house spotted arrays (from the Stanford microarray database) | Integrated co-expression links from 12 GEO ([4](#_ENREF_4)) sets of experiments using the Affymetrix DNA-chip (GPL200) (GSE11055, 12298, 16050, 19310, 2180, 23528, 25633, 32339, 35354, 6547, 8462, 9682) |
| CE-GN | Used a probability-based method only | Integrated links using both the distance-based method and the probability-based method ([5](#_ENREF_5)) |
| CE-GT | Used 2,224 experimentally identified genetic interactions directly from WormBase170 (may include many between-pathway links) | Inferred within-pathway links using the similarity of profiles with 5,780 genetic interactions from WormBase220 |
| CE-HT | PPI by Interactome 5([6](#_ENREF_6)) (represented as CE-YH for v2) | PPI by Interactome 8 ([7](#_ENREF_7)) |
| CE-LC | BIND ([8](#_ENREF_8)), IntAct ([9](#_ENREF_9)), and MINT ([10](#_ENREF_10)) | BIND ([8](#_ENREF_8)), IntAct ([9](#_ENREF_9)), MINT ([10](#_ENREF_10)), BioGrid ([11](#_ENREF_11)), and DIP ([12](#_ENREF_12)) |
| CE-PG | Inferred links from the phylogenetic profiles of 424 bacterial genomes | Integrated links from the profiles of 122 Archaea genomes and 1626 bacterial genomes |
| DM-CX | Not used | Integrated associalogs from the co-expression networks of 30 GEO sets (GSE10012, 11695, 14517, 14531, 16152, 16713, 17013, 17874, 21520, 24978, 27345, 2863, 3057, 33779, 33801, 34400, 3854, 42255, 46550, 47176, 48997, 5430, 7159, 7614, 7763, 8751, 14779, 27163, 33100, 8892) |
| DM-HT | Not used | Integrated associalogs from physical interactions from iRefWeb ([13](#_ENREF_13)) |
| DM-LC | Represented as DM-PI for v2 | Integrated associalogs from physical interactions from iRefWeb ([13](#_ENREF_13)) |
| DR-CX | Not used | Integrated associalogs from the co-expression networks of 21 GEO sets (GSE10188, 11107, 11893, 12991, 13068, 13371, 14495, 14979, 16264, 16740, 17949, 19754, 24528, 32360, 33981, 39731, 4201, 47039, 48806, 8856, 9020) |
| HS-CX | Associalogs from the integrated co-expression network (HS-CX) of HumanNet ([14](#_ENREF_14)) | Integrated associalogs from the individual co-expression networks of 50 GEO sets (GSE10327, 10445, 11903, 12662, 13355, 13425, 14034, 14062, 14209, 14323, 14994, 15935, 16015, 16131, 16214, 16476, 17356, 17700, 17855, 17967, 18723, 19577, 20910, 21122, 2113, 24427, 26366, 26713, 27155, 28497, 29354, 3218, 3307, 34211, 34620, 34733, 36701, 39411, 5847, 6365, 6477, 6740, 7390, 8052, 8218, 8401, 9419, 9874, 9891) |
| HS-HT | Integrated associalogs from affinity purification mass spectrometry analysis data (HS-MS) and high throughput yeast two hybrid data (HS-YH) | Integrated associalogs of high-throughput physical interactions from 6 literature sources ([15-20](#_ENREF_15)) |
| HS-LC | Associalogs from physical interaction DBs (HPRD ([21](#_ENREF_21)), BIND ([8](#_ENREF_8)), BioGRID ([11](#_ENREF_11)), IntAct ([9](#_ENREF_9)), MINT ([10](#_ENREF_10))) | Associalogs from the integrated physical interactions from HPRD ([21](#_ENREF_21)) BioGrid ([11](#_ENREF_11)), , IntAct ([9](#_ENREF_9)), MINT ([10](#_ENREF_10)), DIP ([12](#_ENREF_12)), iRefWeb ([13](#_ENREF_13)) |
| SC-CC | Associalogs from SC-CC of YeastNet v2 ([22](#_ENREF_22)) | Associalogs from SC-CC of YeastNet v3 ([23](#_ENREF_23)) |
| SC-CX | Associalogs from the integrated co-expression network (SC-CX) of YeastNet v2 ([22](#_ENREF_22)) | Integrated associalogs from the individual co-expression networks from 6 Stanford microarray database sets (Cell cycle, DNA damage, Diauxic, Nutrition, Osmotic stress, and YPD stationary growth) and 40 GEO sets (GSE10031, 12220, 12221, 12442, 13684, 14748, 15254, 15936, 16799, 1693, 17364, 17877, 19213, 1934, 20108, 22269, 22832, 23012, 23204, 24802, 24888, 25582, 26829, 26923, 27062, 27235, 30052, 30054, 3076, 31774, 32974, 33276, 33427, 34964, 38848, 40399, 40817, 7645, 8799, 9320) |
| SC-GT | Associalogs from the SC-GT of YeastNet v2 ([22](#_ENREF_22)) | Associalogs from the SC-GT of YeastNet v3 ([23](#_ENREF_23)) |
| SC-HT | Associalogs from links by affinity purification mass spectrometry analysis data (represented as SC-MS of v2). High throughput yeast two hybrid data was excluded due to the low LLS for worm co-functional links. | Associalogs from the SC-HT of YeastNet v3 ([23](#_ENREF_23)) |
| SC-LC | Associalogs from the SC-HT of YeastNet v2 ([22](#_ENREF_22)) | Associalogs from the SC-HT of YeastNet v3 ([23](#_ENREF_23)) |
| SC-TS | Associalogs from the SC-HT of YeastNet v2 ([22](#_ENREF_22)) | Associalogs from the SC-HT of YeastNet v3 (links are the same as for v2) ([23](#_ENREF_23)) |
| CE-CC | Included | Excluded to avoid circular logic |
| HS-CC  HS-DC SC-DC | Included | Excluded due to the bad quality of the new training data |

Data sets are named as XX-YY, where XX represents the species from which the data originated (CE, *Caenorhabditis. elegans*; DM, *Drosophila melanogaster*; DR, *Danio rerio*; HS, *Homo sapiens*; SC, *Saccharomyces cerevisiae*) and YY represents the data type (CC, inferred links from co-citation; CX, inferred links from mRNA co-expression; DC, inferred links from domain co-occurrence; GN, inferred links from gene neighborhood; GT, inferred links from genetic interaction; HT, high-throughput protein-protein interactions; LC, literature-curated protein-protein interactions; PG, inferred links from phylogenetic profile similarity; TS, tertiary structure based protein-protein interactions).

**References**

1. Yook, K., Harris, T.W., Bieri, T., Cabunoc, A., Chan, J., Chen, W.J., Davis, P., de la Cruz, N., Duong, A., Fang, R. *et al.* (2012) WormBase 2012: more genomes, more data, new website. *Nucleic acids research*, **40**, D735-741.

<http://www.ncbi.nlm.nih.gov/pubmed/22067452>

<http://dx.doi.org/10.1093/nar/gkr954>

2. Ashburner, M., Ball, C.A., Blake, J.A., Botstein, D., Butler, H., Cherry, J.M., Davis, A.P., Dolinski, K., Dwight, S.S., Eppig, J.T. *et al.* (2000) Gene ontology: tool for the unification of biology. The Gene Ontology Consortium. *Nature genetics*, **25**, 25-29.

<http://www.ncbi.nlm.nih.gov/pubmed/10802651>

<http://dx.doi.org/10.1038/75556>

3. Kanehisa, M. and Goto, S. (2000) KEGG: kyoto encyclopedia of genes and genomes. *Nucleic acids research*, **28**, 27-30.

<http://www.ncbi.nlm.nih.gov/pubmed/10592173>

4. Barrett, T., Wilhite, S.E., Ledoux, P., Evangelista, C., Kim, I.F., Tomashevsky, M., Marshall, K.A., Phillippy, K.H., Sherman, P.M., Holko, M. *et al.* (2013) NCBI GEO: archive for functional genomics data sets--update. *Nucleic acids research*, **41**, D991-995.

<http://www.ncbi.nlm.nih.gov/pubmed/23193258>

<http://dx.doi.org/10.1093/nar/gks1193>

5. Shin, J., Lee, T., Kim, H. and Lee, I. (2014) Complementarity between distance- and probability-based methods of gene neighbourhood identification for pathway reconstruction. *Molecular bioSystems*, **10**, 24-29.

<http://www.ncbi.nlm.nih.gov/pubmed/24194096>

<http://dx.doi.org/10.1039/c3mb70366e>

6. Li, S., Armstrong, C.M., Bertin, N., Ge, H., Milstein, S., Boxem, M., Vidalain, P.O., Han, J.D., Chesneau, A., Hao, T. *et al.* (2004) A map of the interactome network of the metazoan C. elegans. *Science*, **303**, 540-543.

<http://www.ncbi.nlm.nih.gov/pubmed/14704431>

<http://dx.doi.org/10.1126/science.1091403>

7. Simonis, N., Rual, J.F., Carvunis, A.R., Tasan, M., Lemmens, I., Hirozane-Kishikawa, T., Hao, T., Sahalie, J.M., Venkatesan, K., Gebreab, F. *et al.* (2009) Empirically controlled mapping of the Caenorhabditis elegans protein-protein interactome network. *Nature methods*, **6**, 47-54.

<http://www.ncbi.nlm.nih.gov/pubmed/19123269>

8. Alfarano, C., Andrade, C.E., Anthony, K., Bahroos, N., Bajec, M., Bantoft, K., Betel, D., Bobechko, B., Boutilier, K., Burgess, E. *et al.* (2005) The Biomolecular Interaction Network Database and related tools 2005 update. *Nucleic acids research*, **33**, D418-424.

<http://www.ncbi.nlm.nih.gov/pubmed/15608229>

<http://dx.doi.org/10.1093/nar/gki051>

9. Kerrien, S., Alam-Faruque, Y., Aranda, B., Bancarz, I., Bridge, A., Derow, C., Dimmer, E., Feuermann, M., Friedrichsen, A., Huntley, R. *et al.* (2007) IntAct--open source resource for molecular interaction data. *Nucleic acids research*, **35**, D561-565.

<http://www.ncbi.nlm.nih.gov/pubmed/17145710>

<http://dx.doi.org/10.1093/nar/gkl958>

10. Chatr-aryamontri, A., Ceol, A., Palazzi, L.M., Nardelli, G., Schneider, M.V., Castagnoli, L. and Cesareni, G. (2007) MINT: the Molecular INTeraction database. *Nucleic acids research*, **35**, D572-574.

<http://www.ncbi.nlm.nih.gov/pubmed/17135203>

<http://dx.doi.org/10.1093/nar/gkl950>

11. Stark, C., Breitkreutz, B.J., Reguly, T., Boucher, L., Breitkreutz, A. and Tyers, M. (2006) BioGRID: a general repository for interaction datasets. *Nucleic acids research*, **34**, D535-539.

<http://www.ncbi.nlm.nih.gov/pubmed/16381927>

<http://dx.doi.org/10.1093/nar/gkj109>

12. Salwinski, L., Miller, C.S., Smith, A.J., Pettit, F.K., Bowie, J.U. and Eisenberg, D. (2004) The Database of Interacting Proteins: 2004 update. *Nucleic acids research*, **32**, D449-451.

<http://www.ncbi.nlm.nih.gov/pubmed/14681454>

<http://dx.doi.org/10.1093/nar/gkh086>

13. Turner, B., Razick, S., Turinsky, A.L., Vlasblom, J., Crowdy, E.K., Cho, E., Morrison, K., Donaldson, I.M. and Wodak, S.J. (2010) iRefWeb: interactive analysis of consolidated protein interaction data and their supporting evidence. *Database (Oxford)*, **2010**, baq023.

<http://www.ncbi.nlm.nih.gov/pubmed/20940177>

<http://dx.doi.org/10.1093/database/baq023>

14. Lee, I., Blom, U.M., Wang, P.I., Shim, J.E. and Marcotte, E.M. (2011) Prioritizing candidate disease genes by network-based boosting of genome-wide association data. *Genome research*, **21**, 1109-1121.

<http://www.ncbi.nlm.nih.gov/pubmed/21536720>

<http://dx.doi.org/10.1101/gr.118992.110>

15. Sowa, M.E., Bennett, E.J., Gygi, S.P. and Harper, J.W. (2009) Defining the human deubiquitinating enzyme interaction landscape. *Cell*, **138**, 389-403.

<http://www.ncbi.nlm.nih.gov/pubmed/19615732>

<http://dx.doi.org/10.1016/j.cell.2009.04.042>

16. Havugimana, P.C., Hart, G.T., Nepusz, T., Yang, H., Turinsky, A.L., Li, Z., Wang, P.I., Boutz, D.R., Fong, V., Phanse, S. *et al.* (2012) A census of human soluble protein complexes. *Cell*, **150**, 1068-1081.

<http://www.ncbi.nlm.nih.gov/pubmed/22939629>

<http://dx.doi.org/10.1016/j.cell.2012.08.011>

17. Yu, H., Tardivo, L., Tam, S., Weiner, E., Gebreab, F., Fan, C., Svrzikapa, N., Hirozane-Kishikawa, T., Rietman, E., Yang, X. *et al.* (2011) Next-generation sequencing to generate interactome datasets. *Nature methods*, **8**, 478-480.

<http://www.ncbi.nlm.nih.gov/pubmed/21516116>

<http://dx.doi.org/10.1038/nmeth.1597>

18. Wang, J., Huo, K., Ma, L., Tang, L., Li, D., Huang, X., Yuan, Y., Li, C., Wang, W., Guan, W. *et al.* (2011) Toward an understanding of the protein interaction network of the human liver. *Mol Syst Biol*, **7**, 536.

<http://www.ncbi.nlm.nih.gov/pubmed/21988832>

<http://dx.doi.org/10.1038/msb.2011.67>

19. Ewing, R.M., Chu, P., Elisma, F., Li, H., Taylor, P., Climie, S., McBroom-Cerajewski, L., Robinson, M.D., O'Connor, L., Li, M. *et al.* (2007) Large-scale mapping of human protein-protein interactions by mass spectrometry. *Mol Syst Biol*, **3**, 89.

<http://www.ncbi.nlm.nih.gov/pubmed/17353931>

<http://dx.doi.org/10.1038/msb4100134>

20. Hutchins, J.R., Toyoda, Y., Hegemann, B., Poser, I., Heriche, J.K., Sykora, M.M., Augsburg, M., Hudecz, O., Buschhorn, B.A., Bulkescher, J. *et al.* (2010) Systematic analysis of human protein complexes identifies chromosome segregation proteins. *Science*, **328**, 593-599.

<http://www.ncbi.nlm.nih.gov/pubmed/20360068>

<http://dx.doi.org/10.1126/science.1181348>

21. Keshava Prasad, T.S., Goel, R., Kandasamy, K., Keerthikumar, S., Kumar, S., Mathivanan, S., Telikicherla, D., Raju, R., Shafreen, B., Venugopal, A. *et al.* (2009) Human Protein Reference Database--2009 update. *Nucleic acids research*, **37**, D767-772.

<http://www.ncbi.nlm.nih.gov/pubmed/18988627>

<http://dx.doi.org/10.1093/nar/gkn892>

22. Lee, I., Li, Z. and Marcotte, E.M. (2007) An improved, bias-reduced probabilistic functional gene network of baker's yeast, Saccharomyces cerevisiae. *PLoS One*, **2**, e988.

<http://www.ncbi.nlm.nih.gov/pubmed/17912365>

<http://dx.doi.org/10.1371/journal.pone.0000988>

23. Kim, H., Shin, J., Kim, E., Kim, H., Hwang, S., Shim, J.E. and Lee, I. (2014) YeastNet v3: a public database of data-specific and integrated functional gene networks for Saccharomyces cerevisiae. *Nucleic acids research*, **42**, D731-736.

<http://www.ncbi.nlm.nih.gov/pubmed/24165882>

<http://dx.doi.org/10.1093/nar/gkt981>
